# Tweenager Computer Visual Syndrome Due to Tablets and Laptops during the Postlockdown COVID-19 Pandemic and the Influence on the Binocular and Accommodative System

**DOI:** 10.3390/jcm11185317

**Published:** 2022-09-09

**Authors:** Concepción De-Hita-Cantalejo, José-María Sánchez-González, Carmen Silva-Viguera, María Carmen Sánchez-González

**Affiliations:** Department of Physics of Condensed Matter, Optics Area, University of Seville, 41012 Seville, Spain

**Keywords:** computer vision syndrome, accommodative disorders, binocular vision disorders, preteens, smartphones, tables, digital devices

## Abstract

The aim of our study was to compare computer visual syndrome (CVS) in a tweenager student population who use tablets and laptops only to play versus CVS in tweenagers who use these digital devices at school to study, in addition to playing. The tests performed were a validated survey for children for the detection of CVS and accommodative and vergence tests. The CVS item questionnaire was divided into four main groups based on questions concerning the following: (I) the digital device usage time, (II) musculoskeletal and ergonomic nature, (III) visual symptoms, and (IV) ocular surface symptoms. The high-demand digital device group showed worse punctuation in all item groups. From the optometric perspective, when the subjects were classified according to the CVS, high-demand participants presented a clear tendency to exophoria with statistically significant differences in distance vision (−1.94 ± 4.48 Δ) and near vision (−5.78 ± 8.62 Δ) (*p* < 0.01). Our results establish a relationship between the increased use of electronic devices and computer vision syndrome in the preadolescent population. In addition, this situation is related to the presence of visual, accommodative, and binocular dysfunctions that could affect the efficiency of the visual system.

## 1. Introduction

In 2020, Spain suffered a mandatory lockdown of eight weeks due to the pandemic caused by the coronavirus disease (COVID-19) [1]. This situation forced adults and children to change their work and study habits; adults began to telework from home, while children received their teaching through digital devices [2]. At the end of the lockdown, the use of digital devices in the day-to-day of some schools had increased due to successive contagions and the absences of students from the classrooms. Consequently, the digital device screen use time by children increased, which can lead to an increase in all symptoms associated with the abuse of these devices [3,4], defined as computer visual syndrome (CVS).

The American Optometric Association defined CVS as “a set of ocular and visual signs and symptoms related to the use of electronic devices for a long time” [5]. Head, neck, and shoulder pain must be added to this symptomatology [6]. Although few studies have examined the effect of CVS on children, the symptomatology in children was observed to be similar to that of adults; however, these devices more severely damage children than adults [7] because children continuously use these devices at school and in their leisure hours [8]. Specifically, the symptomatology is exacerbated in children because of the following [9]: (1) the abuse of digital screens due to a lack of awareness because most children do not take the due breaks, especially when they are playing, and they play for many hours in a row, which can cause both accommodative and binocular disorders; (2) the use of computers by adults; since most children use computers at the table where an adult does, their binocular vision is altered by a change in the focusing distance. Furthermore, they experience pain in the arms, neck, and back due to improper posture to use the keyboard; and (3) poor lighting, which can lead to glare and focusing problems if the light level is higher. With regard to accommodative and binocular changes, regulatory values are affected by the use of digital devices; at the same time, these changes increase ocular symptomatology [10].

The accommodative and binocular systems work together, and if these fail due to overwork in close proximity, accommodative and/or nonstrabismic binocular alterations will occur, which can produce different asthenopics symptoms [11]. From the perspective of refractive errors, the prolonged use of close work without scheduled breaks increases the prevalence of myopia among students [12].

Therefore, the aim of our study was to compare CVS in a tweenager population of students who use tablets and laptops only to play versus adolescents who use these digital devices at school to study, in addition to playing.

## 2. Materials and Methods

This prospective, descriptive, and cross-sectional study was conducted in February 2022. Students from the 5th and 6th grades of primary school, with ages ranging from 10 to 12 years, were recruited. The tests performed were a validated survey for children for the detection of CVS, visual acuity (VA) measurement with or without correction depending on the case, cover test, accommodative tests, and vergence tests. The data obtained from the survey and the values obtained from the optometric measurements were correlated.

### 2.1. Ethical Aspects

Before starting the measurements, an informative email was sent to the parents explaining all the tests to be conducted, and an informed consent signed and returned, authorizing participation in the study. Consent was signed before initiating measurements.

This study was conducted in accordance with the Declaration of Helsinki, and the Institutional Review Board of the University Hospital Virgen Macarena of the University of Seville approved the research.

### 2.2. Patients

The following participants were included: (1) students in the 5th and 6th grades of primary school and (2) students who answered the complete CVS survey. The following participants were excluded: (1) participants who did not deliver the informed consent document signed by parents or guardians, (2) participants having an ocular or systemic pathology that could alter the data of the visual tests, (3) participants taking drugs that could modify the data obtained, (4) participants that had undergone eye surgery, and (5) participants suffering from binocular problems, phoria, or tropia, and (6) refraction over 4 diopters of myopia, hyperopia, and astigmatism [13]. Of a total of 122 children, 108 met the inclusion criteria, and 14 were excluded from the study, all for not providing signed parental consent.

The students were divided into two groups, those with a high demand, who studied and played with digital devices; and those with low demand, who only used them to play.

### 2.3. Material and Measures

The material used was a validated CVS screening questionnaire for children [14,15], ±2.00 D flippers, near-vision card, opaque occluder, translucent occluder, tape measure, prism bars, pointer (all previous material from Optometric Promotion, Madrid, Spain), Welch Allyn Streak Retinoscope (Welch Allyn, Leicestershire, UK), and Welch Allyn Autorefractometer (Welch Allyn, Leicestershire, UK). The measurements were made under the same light conditions and with the same material for all children. Before beginning to answer the questionnaire and conducting each optometric test, the procedure to be conducted was explained. If the method was not clear, the explanation was repeated until the measurements were taken correctly. The questionnaire used to assess the CVS was based on a questionnaire already validated for children [15], which was given to teachers to complete in the classroom. At the time of filling in the questionnaire, an optometrist went to the classroom to explain how to proceed and resolve doubts when answering the questions.

In conducting the optometric tests, the most repeatable method that allowed taking the measurements with mobile equipment was selected. All measurements were taken with the correction of the child, who was seated with feet on the ground. Visual acuity (VA) in mono- and binocular far vision was tested with an Early Treatment Diabetic Retinopathy Study (ETDRS) card optimized for a distance of 4 m [16]. The mono- and binocular amplitude of accommodation (AA) was measured using the distance method, which is the most repeatable method for portable equipment [17]. Accommodative posture (AP) was measured by Nott dynamic retinoscopy using a close-up optotype as an accommodative stimulus located at 40 cm and under binocular conditions. To calculate the accommodative posture, the accommodative stimulus in D ($\frac{1}{40\times 10^{-2}}$) was subtracted from the accommodative response in D ($\frac{1}{Retinoscope\;Distance\times 10^{-2}}$) [18]. To measure accommodative flexibility (AF), a flipper of ±2.00 D and an optotype close to 40 cm from the patient were used [19]. If we observed that the child had trouble seeing the letters with positive and/or negative lenses during the test, we noted this behavior as the final result. The near point of convergence (NPC) was measured under binocular conditions using a pointer as an accommodative stimulus [20]. To align visual axes, the cover test (CT) and alternate cover test (CA) were used, following the criterion of greater repeatability [21]. Fusional vergences (FVs) were measured by jumping with a prism bar because this method could be performed with portable equipment [20]. For normative values, we relied on the manual by Scheiman and Wick [20].

## 3. Results

One hundred and eight tweenagers between 10 and 12 years old were included in this case-control study. Forty male preteens (37%) and sixty-eight female preteens (63%) comprised the study sample. The frequency, mean age, nineteen CVS questionnaire items, and mean CVS questionnaire value differences between the low- and high-demand digital device groups are presented in Table 1. Regarding the frequency of days per week in the low-demand and high-demand group, 61.0% and 26.5% used digital devices from 1 to 3 days per week, 18.6% and 44.69% used digital devices from 4 to 6 days per week, and 20.3% and 28.6% used them all days, respectively. Regarding hours per day in the low-demand and high-demand groups, 13.6% and 53.1% used digital devices for more than 5 h per day, 42.4% and 34.7% used them for between 3 and 4 h, 18.6% and 12.2% used them for between 1 and 2 h, and 25.4% and 0.0% used them for less than 1 h, respectively. The decimal scale visual acuity, accommodation amplitude, accommodative posture, and facility differences between the low- and high-demand digital device groups are shown in Table 2. Finally, within the quantitative analysis, phoria, near point of convergence, and negative and positive fusional vergence variations among both digital device groups are described in Table 3.

The CVS item questionnaire was divided into four main groups based on questions concerning the following [15,22,23]: (I) items on digital device usage time, (II) items on musculoskeletal and ergonomic nature, (III) items on visual symptoms, and (IV) items on ocular surface symptoms. The high-demand digital device group achieved worse punctuation in all item groups. The low- and high-demand digital device groups achieved 2.45 ± 0.49 and 3.04 ± 0.57 score points, respectively, on the usage time item (*p* < 0.01). The low- and high-demand digital device groups reached 2.49 ± 0.47 and 3.10 ± 0.45 score points on musculoskeletal and ergonomic items, respectively (*p* < 0.01). The low- and high-demand digital device groups scored 2.97 ± 0.34 and 3.59 ± 0.35 score points, respectively, on the visual symptoms item (*p* < 0.01). Finally, the low- and high-demand groups achieved scores of 2.15 ± 0.35 and 3.22 ± 0.31, respectively, on the ocular surface item (*p* < 0.01).

**Figure 1:**
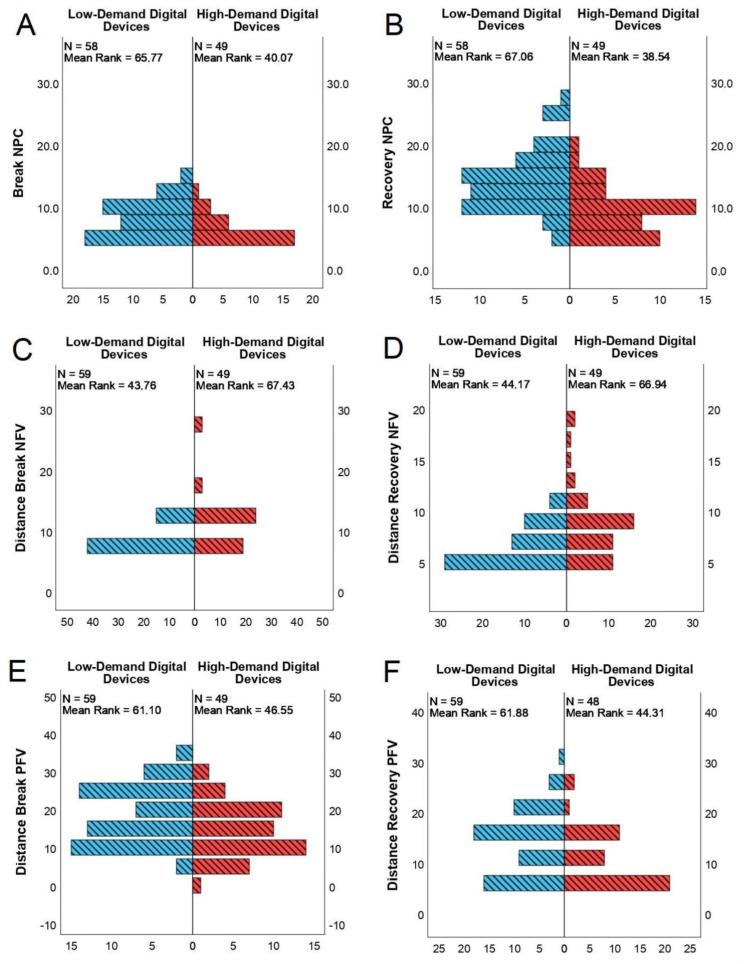
The population pyramids between both low- and high-demand groups. (**A**) Break near point of convergence (centimeters), (**B**) recovery near point of convergence (centimeters), (**C**) distance break negative fusional vergence (prism diopter), (**D**) distance recovery negative fusional vergence (prism diopter), (**E**) distance break positive fusional vergence (prism diopter), and (**F**) distance recovery positive fusional vergence (prism diopter).

Regarding statistically significant differences between the low- and high-demand digital device groups, the following statistical value and size effects were found: right (U = 15.00), left (U = 9.00), and both eyes (U = 0.00) visual acuity, with exceptionally large size effects (3.89, 4.13, and 9.71, respectively). Near (U = 939.00) and distance phoria, with moderate size effects (0.70 and 0.50, respectively) were also observed. Break (U = 738.50) and recovery (U = 663.50) NPCs, with large size effects (0.88 and 0.90, respectively) were noted. Break (U = 2079.00) and recovery (U = 2055.00) NFVs with large size effects (0.85 and 0.82, respectively) were recorded. Finally, break (U = 1056.00) and recovery (951.00) PFVs were observed, with moderate size effects (0.54 and 0.59, respectively). The population pyramids between both low- and high-demand groups within break and recovery NPCs, distance break and recovery NFVs, and distance break and recovery PFVs are shown in Figure 1. No statistically or clinically significant strong correlations were identified.

## 4. Discussion

This observational, descriptive, and cross-sectional study analyzed CVS in children between 10 and 12 years of age through a validated survey for children [15]. In addition, optometric variables, such as CT, CA, VA, AA, AP, AF, NPC, and FV, were analyzed to determine possible binocular and accommodative alterations associated with the use of digital devices. According to the results of the survey, 49 preadolescents of the total sample analyzed were part of the high-CVS group (49% boys and 51% girls) and 59 were part of the low-CVS group (27.1% boys and 72.9% girls). The 19 items of the survey are grouped into four groups: Group 1 included the time of use of digital devices, Group 2 referred to musculoskeletal symptoms and ergonomics, Group 3 analyzed visual symptoms, and Group 4 analyzed alterations of the eye surface.

In the first group, which examined the time of use, the high-demand group obtained a worse score (3.04 ± 0.57) than the “low-demand” group (2.45 ± 0.49). This situation corresponds to that reported by several studies that related a longer exposure time to digital devices with an increase in general symptoms [24,25]; specifically, Mohan et al. [2] conducted a study in which they observed an increase in digital eye strain in children in the COVID-19 era due to increased time spent using digital devices. In the second group, which examined musculoskeletal symptoms and ergonomics, including headache and neck and back pain, the high-demand group also obtained a worse score (2.49 ± 0.47) than the “low-demand” group (3.10 ± 0.45); similarly, Galindo-Romero et al. [1] conducted a study of 730 patients between the ages of 18 and 73 in which they evaluated the symptomatology related to CVS during confinement due to COVID-19, concluding that 36.7% of the participants suffered from headache. In addition, they noted that the symptoms were more pronounced in younger participants, especially in participants between 18 and 30 years old. Although our population consisted of adolescents, their findings corroborate the data of our study.

Moreover, Leng et al. [26] carried out a study in 40 elementary schools in which they assessed the physical problems in children due to the prolonged use of digital devices, concluding that 32.9% of children suffered from headache and neck and back pain. Both studies and those of other authors also refer to headache and neck and back pain due to the prolonged use of these devices in children [27,28]. In the third group, we analyzed the visual symptomatology. To this end, Ekemiri et al. [29] conducted a study in which they analyzed the increase in eye discomfort in 435 schoolchildren, which was associated with an increase in the time of use of digital devices during confinement due to COVID-19. Specifically, 65.1% of the schoolchildren reported blurred vision, 33.5% reported double vision, and 75% also reported headache.

In our study, students with a high demand for the use of digital devices obtained a worse score in this group of symptoms (2.97 ± 0.34) than the low-demand students (3.59 ± 0.35). This increase in symptoms is a consequence of the increase in the use of electronic devices, which corresponds to the effects described by other authors [30,31]. Finally, the fourth group, which examined alterations of the ocular surface, also showed worse results in the high-demand group (2.15 ± 0.35) than the “low-demand” group (3.22 ± 0.31). Our results are consistent with those of authors who established a relationship between ocular dryness and the use of digital devices [24,32]. Golebiowski et al. [33] carried out a study of young people without dry eye, measuring changes in the ocular surface before and after 60 min of reading with digital devices. They reported increasing ocular surface symptoms after reading and observed a decrease in the blinking time associated with this increased symptomatology.

From the optometric perspective, when the subjects were classified according to the CVS, the high-demand participants presented a clear tendency toward exophoria, with statistically significant differences in distance vision (−1.94 ± 4.48 Δ) and near vision (−5.78 ± 8.62 Δ). This sign could be associated with the presence of convergence insufficiency [34,35,36]. In addition, the values of the variables “distance break NFV” (10.96 ± 4.73 Δ) and “distance recovery NFV” (7.71 ± 3.44 Δ) were higher than the norm in this group of participants, which causes a tendency toward greater amplitudes of divergence and is associated with exodeviation [37,38,39]. Similarly, the variables “distance break PFV” and “distance recovery PFV” in the high-demand group showed significantly decreased values (14.08 ± 6.95 Δ) and (9.73 ± 5.66 Δ), respectively, compared to the “low-demand” group. This situation could determine altered horizontal fusion amplitude ranges [35,40]. Regarding NPC, the results are not conclusive, since the high-demand group presented significantly closer values than those of the “low-demand” group. Most authors use the measurement of deviation to define the state of binocular function, but not all consider other tests, such as NPC, to diagnose convergence insufficiency. Therefore, we considered the value of phoria, the main clinical sign to determine the binocular status of the participants, and other clinical signs were considered to be complementary (Table 3, Figure 1).

Nonstrabismic binocular dysfunctions are frequent visual alterations, a consequence of prolonged visual demand at close distances. Alterations in the ranges of horizontal vergences could be a consequence of the overexertion of the extraocular muscles because of the continued use of electronic devices. These dysfunctions cause diverse symptoms that include asthenopia and difficulty in efficiently performing certain activities that require a greater demand for near vision [11,41] and can be associated with musculoskeletal discomfort in the neck and shoulder area. This situation is related to the presence of CSV [42].

Similar to other authors [15,43], our research shows the presence of an exodeviation after the uninterrupted use of electronic devices, which causes an overexertion in vergential function, altering the efficiency of the visual system and accentuating CVS symptoms. Additionally, in our results, we observed alterations in the ranges of horizontal fusional vergence in the high-demand group. Zhang et al. [44] describe decreased fusional vergence in both directions, convergence and divergence, in a group of children while watching television. These children displayed abnormal head posture, probably to compensate for this deficiency. An inefficient visual system can increase musculoskeletal discomfort [45] and the symptomatology of CVS [15,46], as our results show.

This study had some limitations. First, the time of use of digital devices should be controlled with an objective method; control via the survey may cause the child to feel controlled and not answer with total sincerity and task type track should be implemented in future research. Second, no follow-up was conducted to assess the evolution of the symptoms or the alterations in the optometric tests. Third, the optometric measurements were made with mobile equipment, and making these measurements in an optometric office with a phoropter may be more accurate for some measurements. Finally, another limitation was the difference between age groups. This difference was statistically significant but not clinically significant. Nevertheless, the use of portable equipment allowed us to conduct the study on a larger number of children. Future research should include refraction or longitudinal axial length to improve the findings.

## 5. Conclusions

The results of our study establish a relationship between the increased use of electronic devices in classrooms after the COVID-19 pandemic and computer vision syndrome in the preadolescent population. In addition, this situation is related to the presence of visual, accommodative, and binocular dysfunctions that could affect the efficiency of the visual system. Optometric clinical screening assessments should be recommended to reduce ocular symptomatology and prevent long-term effects. Parental awareness and training on CVS are essential to promote its prevention.

## Figures and Tables

**Table 1:** Demographics and computer visual syndrome questionnaire characteristics.

Variables (Units)	Low-Demand Digital Devices (*n* = 59)	High-Demand Digital Devices (*n* = 49)	*p* Value
Male (%) Female (%)	16 (27.1) 43 (72.9)	24 (49.0) 25 (51.0)	0.05
Caucasian (%) Asian (%)	57 (96.6) 2 (3.4)	46 (93.8) 3 (6.2)	0.85
Age (years)	10.80 ± 0.76 (10 to 12)	11.02 ± 0.47 (10 to 12)	<0.01
Item Questionnaire 1 (Score Points)	2.44 ± 1.02 (1 to 3)	3.41 ± 0.70 (2 to 4)	<0.01
Item Questionnaire 2 (Score Points)	2.59 ± 0.81 (2 to 4)	3.02 ± 0.75 (2 to 4)	<0.01
Item Questionnaire 3 (Score Points)	2.32 ± 0.53 (2 to 4)	2.69 ± 0.87 (1 to 4)	<0.01
Item Questionnaire 4 (Score Points)	2.42 ± 0.59 (1 to 3)	3.16 ± 0.74 (1 to 4)	<0.01
Item Questionnaire 5 (Score Points)	1.83 ± 0.74 (1 to 3)	3.43 ± 0.67 (2 to 4)	<0.01
Item Questionnaire 6 (Score Points)	2.37 ± 0.82 (1 to 4)	3.16 ± 0.74 (2 to 4)	<0.01
Item Questionnaire 7 (Score Points)	3.02 ± 0.82 (1 to 4)	3.35 ± 0.77 (2 to 4)	0.03
Item Questionnaire 8 (Score Points)	2.36 ± 0.66 (1 to 3)	3.61 ± 0.53 (2 to 4)	<0.01
Item Questionnaire 9 (Score Points)	3.32 ± 0.95 (1 to 4)	3.59 ± 0.61 (1 to 4)	0.26
Item Questionnaire 10 (Score Points)	3.39 ± 0.96 (1 to 4)	3.78 ± 0.51 (2 to 4)	0.02
Item Questionnaire 11 (Score Points)	3.69 ± 0.67 (1 to 4)	3.73 ± 0.56 (1 to 4)	0.97
Item Questionnaire 12 (Score Points)	3.34 ± 0.95 (1 to 4)	3.71 ± 0.50 (2 to 4)	0.05
Item Questionnaire 13 (Score Points)	2.25 ± 0.95 (1 to 4)	3.33 ± 0.77 (1 to 4)	<0.01
Item Questionnaire 14 (Score Points)	2.17± 1.10 (1 to 4)	3.53 ± 0.61 (2 to 4)	<0.01
Item Questionnaire 15 (Score Points)	1.59 ± 0.91 (0 to 4)	3.80 ± 0.40 (3 to 4)	<0.01
Item Questionnaire 16 (Score Points)	1.63 ± 0.96 (1 to 4)	3.65 ± 0.59 (2 to 4)	<0.01
Item Questionnaire 17 (Score Points)	2.53 ± 1.08 (1 to 4)	3.10 ± 0.77 (1 to 4)	<0.01
Item Questionnaire 18 (Score Points)	2.53± 0.93 (1 to 4)	3.06 ± 0.74 (2 to 4)	<0.01
Item Questionnaire 19 (Score Points)	1.93 ± 1.08 (1 to 4)	1.45 ± 0.73 (1 to 4)	0.01
Mean Computer Vision Syndrome (Score Points)	2.52 ± 0.11 (2.42 to 3.05)	3.12 ± 0.28 (2.47 to 3.68)	<0.01
	(2.42 to 3.05)		
Computer Visual Syndrome Survey is shown in Supplementary Document S1			

**Table 2:** Visual acuity and accommodative system between low- and high-demand digital device groups.

Variables (Units)	Low-Demand Digital Devices (*n* = 59)	High-Demand Digital Devices (*n* = 49)	*p* Value
Visual Acuity (Decimal Scale)			
Right Eye	1.11 ± 0.15 (0.80 to 1.25)	0.64 ± 0.07 (0.60 to 0.90)	<0.01
Left Eye	1.11 ± 0.14 (0.80 to 1.25)	0.65 ± 0.06 (0.60 to 0.90)	<0.01
Both Eyes	1.22 ± 0.07 (1.00 to 1.25)	0.62 ± 0.05 (0.60 to 0.90)	<0.01
Accommodation Amplitude			
Right Eye (centimeters)	9.63 ± 2.81 (6.00 to 25.00)	9.05 ± 2.21 (5.00 to 14.00)	0.46
Right Eye (diopters)	11.00 ± 2.44 (4.00 to 16.67)	11.77 ± 3.15 (7.14 to 20.00)	0.46
Left Eye (centimeters)	9.88 ± 2.87 (6.00 to 23.50)	9.01 ± 2.16 (6.00 to 15.00)	0.23
Left Eye (diopters)	10.76 ± 2.42 (4.26 to 16.67)	11.76 ± 2.92 (6.67 to 16.67)	0.23
Both Eyes (centimeters)	9.61 ± 2.58 (4.00 to 19.50)	9.37 ± 3.03 (5.00 to 20.00)	0.62
Both Eyes (diopters)	11.14 ± 3.36 (5.13 to 25.00)	11.66 ± 3.54 (5.00 to 20.00)	0.62
Accommodative Posture and Facility			
Both Eyes Posture (centimeters)	44.56 ± 6.79 (31.00 to 80.00)	48.79 ± 12.98 (30.00 to 100.00)	0.20
Both Eyes Posture (diopters)	0.21 ± 0.31 (−0.73 to 1.25)	0.35 ± 0.40 (−0.83 to 1.50)	0.20
Both Eyes Facility (cycles per minute)	8.50 ± 3.33 (2.00 to 15.00)	9.53 ± 5.19 (0.00 to 21.00)	0.11

**Table 3:** Phoria and vergence systems between low- and high-demand digital device groups.

Variables (Units)	Low-Demand Digital Devices (*n* = 59)	High-Demand Digital Devices (*n* = 49)	*p* Value
Phoria (negative exophoria and positive endophoria) Near Point of Convergence (NPC)			
Near Phoria (Δ, prism diopter)	−1.53 ± 2.07 (−12.00 to 0.00)	−5.78 ± 8.62 (−25.00 to +12.00)	<0.01
Distance Phoria (Δ, prism diopter)	−0.24 ± 2.10 (−16.00 to +2.00)	−1.94 ± 4.48 (−16.00 to +8.00)	<0.01
Break NPC (centimeter)	7.31 ± 3.57 (0.00 to 15.00)	4.35 ± 3.05 (0.00 to 12.50)	<0.01
Recovery NPC (centimeter)	13.01 ± 5.91 (0.00 to 27.00)	8.20 ± 4.52 (0.00 to 19.00)	<0.01
Negative Fusional Vergences (NFVs)			
Near Break NFV (Δ, prism diopter)	11.02 ± 2.81 (4.00 to 16.00)	11.76 ± 5.98 (2.00 to 35.00)	0.90
Near Recovery NFV (Δ, prism diopter)	8.14 ± 2.62 (2.00 to 14.00)	8.10 ± 4.84 (1.00 to 20.00)	0.35
Distance Break NFV (Δ, prism diopter)	7.83 ± 2.47 (2.00 to 14.00)	10.96 ± 4.73 (6.00 to 25.00)	<0.01
Distance Recovery NFV (Δ, prism diopter)	5.41 ± 2.10 (1.00 to 10.00)	7.71 ± 3.44 (4.00 to 18.00)	<0.01
Positive Fusional Vergences (PFVs)			
Near Break PFV (Δ, prism diopter)	14.29 ± 6.45 (2.00 to 30.00)	14.96 ± 7.57 (0.00 to 30.00)	0.65
Near Recovery PFV (Δ, prism diopter)	10.76 ± 5.63 (1.00 to 25.00)	10.70 ± 5.01 (1.00 to 20.00)	0.80
Distance Break PFV (Δ, prism diopter)	18.22 ± 8.09 (4.00 to 35.00)	14.08 ± 6.95 (0.00 to 30.00)	<0.01
Distance Recovery PFV (Δ, prism diopter)	13.31 ± 6.37 (2.00 to 30.00)	9.73 ± 5.66 (1.00 to 25.00)	<0.01

## Data Availability

The data presented in this study are available on request from the corresponding author. The data are not publicly available due to their containing information that could compromise the privacy of research participants.

## Supplementary Materials

The following supporting information can be downloaded at: https://www.mdpi.com/article/10.3390/jcm11185317/s1, Document S1: Computer Visual Syndrome Survey.
