# Supplementary material for: Tweenager Computer Visual Syndrome Due to Tablets and Laptops during the Postlockdown COVID-19 Pandemic and the Influence on the Binocular and Accommodative System
Source: J Clin Med. 2022 Sep 9;11(18):5317. doi: 10.3390/jcm11185317 (PMC9501452; doi:10.3390/jcm11185317)
Supplement: Supplementary file 1 [file jcm-11-05317-s001.zip › jcm-1888529-supplementary.pdf]

Computer Visual Syndrome Survey<br>Name:<br>Age:<br>Sex:	UNIVERSIDAD Ð SEVILLA

1.Do you use digital devices to study?			
Never	Sometimes	Often	Always
2. How many days per week do you play/study with digital screens?			
1 day or less	From 1 to 3 days	From 4 to 6 days	All days
3. How many hours per day do you play/study with digital screens?			
1 hours or less	From 1 to 2 hours	From 3 to 4 hours	More than 5 hours
4. Do you use digital devices with the light off?			
Never	Sometimes	Often	Always
5. Have you noticed that sometimes you get blurred letters on digital screens while playing/studying with them?			
Never	Sometimes	Often	Always
6. Do you notice your eyes tired during or after using computers or tablets?			
Never	Sometimes	Often	Always
7. Have you noticed that your eyes hurt before or after using digital screens?			
Never	Sometimes	Often	Always
8. Have you noticed a burning sensation in your eyes during or after using digital screens?			
Never	Sometimes	Often	Always
9. Have you noticed that, after digital screens you have to strive to be able to see well from afar?			
Never	Sometimes	Often	Always
10. Have you noticed that, after some time with digital screens, you have difficulty reading?			
Never	Sometimes	Often	Always
11. While using digital screens, do you have the feeling that you get cross-eyed?			
Never	Sometimes	Often	Always
12. Have you noticed that when you spend a lot of time with the computer there comes a time when you end up seeing the double letters?			
Never	Sometimes	Often	Always
13.Have you noticed that, after some time with the computer, you are bothered by the lights?			
Never	Sometimes	Often	Always
14. In recent weeks have you noticed watery eyes during or after the use of digital screens?			
Never	Sometimes	Often	Always
15. In recent weeks have you noticed dry eyes?			
Never	Sometimes	Often	Always
16. In recent weeks have you noticed red eyes?			
Never	Sometimes	Often	Always
17. Has your headache after using electronic devices?			
Never	Sometimes	Often	Always
18. Do you feel head/back/neck pain after using electronic devices?			
Never	Sometimes	Often	Always
19. If you have suffered some of these symptoms, Does it disappear?			
Never	Sometimes	Often	Always
